# Empirical validation of the UNAIDS Spectrum model for subnational HIV estimates: case-study of children and adults in Manicaland, Zimbabwe

**DOI:** 10.1097/QAD.0000000000001418

**Published:** 2017-04

**Authors:** Romain Silhol, Simon Gregson, Constance Nyamukapa, Mutsa Mhangara, Janet Dzangare, Elizabeth Gonese, Jeffrey W. Eaton, Kelsey K. Case, Mary Mahy, John Stover, Owen Mugurungi

**Affiliations:** a Department of Infectious Disease Epidemiology, Imperial College London, London, UK; b Biomedical Research and Training Institute, Avondale, Harare, Zimbabwe; c AIDS and TB Unit, Zimbabwe Ministry of Health and Child Welfare, Harare, Zimbabwe; d Division of Global HIV and TB, U.S. Centers for Disease Control and Prevention, Harare, Zimbabwe; e Programme Branch, UNAIDS, Geneva, Switzerland; f Avenir Health, Glastonbury, Connecticut, USA.

**Keywords:** HIV estimates, HIV incidence rate, Mathematical model validation, models/projections, paediatric estimates, Spectrum model, Zimbabwe

## Abstract

**Background:**

More cost-effective HIV control may be achieved by targeting geographical areas with high infection rates. The AIDS Impact model of Spectrum –— used routinely to produce national HIV estimates –— could provide the required subnational estimates but is rarely validated with empirical data, even at a national level.

**Design:**

The validity of the Spectrum model estimates were compared with empirical estimates.

**Methods:**

Antenatal surveillance and population survey data from a population HIV cohort study in Manicaland, East Zimbabwe, were input into Spectrum 5.441 to create a simulation representative of the cohort population. Model and empirical estimates were compared for key demographic and epidemiological outcomes. Alternative scenarios for data availability were examined and sensitivity analyses were conducted for model assumptions considered important for subnational estimates.

**Results:**

Spectrum estimates generally agreed with observed data but HIV incidence estimates were higher than empirical estimates, whereas estimates of early age all-cause adult mortality were lower. Child HIV prevalence estimates matched well with the survey prevalence among children. Estimated paternal orphanhood was lower than empirical estimates. Including observations from earlier in the epidemic did not improve the HIV incidence model fit. Migration had little effect on observed discrepancies –— possibly because the model ignores differences in HIV prevalence between migrants and residents.

**Conclusion:**

The Spectrum model, using subnational surveillance and population data, provided reasonable subnational estimates although some discrepancies were noted. Differences in HIV prevalence between migrants and residents may need to be captured in the model if applied to subnational epidemics.

## Introduction

Country teams produce national HIV estimates using Spectrum model simulations [1]. These models require national demographic, HIV prevalence, and programme input data. Historically, HIV prevalence data in generalized HIV epidemics came solely from surveillance of women attending ante-natal clinics (ANC) and were adjusted for known biases [2]. Since 2006, general population prevalence data collected in household surveys are utilized with sentinel surveillance data. Increasing the quantity of locally specific data within the model is believed to improve the accuracy of estimates.

Spectrum files were available for 160 countries in 2016 [3]. Model development is driven by country needs and increasing availability of more detailed data. Despite its global and frequent use, Spectrum model estimates are infrequently compared with empirical data [4–7]. Also, there is debate as to whether the model produces reliable estimates of AIDS mortality [8,9]. The global burden of disease 2015 study, utilizing data on all-cause mortality, recently estimated significantly higher mortality in sub-Saharan Africa than the Joint United Nations Programme on HIV/AIDS (UNAIDS) Spectrum-based estimates for 2014 [10]. Spectrum is also used extensively by countries to measure the performance of their Antiretroviral therapy (ART) and Prevention of Mother-To-Child Transmission (PMTCT) programmes, and improvements in the methods for producing child estimates (HIV prevalence, orphanhood, ART coverage) have been included in the most recent model versions [11].

Recent work has shown that national responses to the HIV epidemic could be improved by targeting populations at a subnational scale [12]. Such an approach would require subnational HIV estimates and Spectrum may be used by country teams to produce these estimates [13,14]. As locally specific data for model inputs will be limited in some areas, it is important to assess the accuracy and the robustness of Spectrum model estimates under these circumstances, and identify which subnational data influenced the HIV estimates obtained. Stakeholders such as the Global Fund or President’s Emergency Plan For AIDS Relief are interested in subnational allocation to improve program cost-effectiveness [3,15,16].

In this study, we address these questions using data from the Manicaland region of eastern Zimbabwe where the population is subject to a generalized HIV epidemic. The study aimed: to apply the Spectrum model using subnational surveillance and survey data, and to validate key outcomes using epidemiological and demographic data from the same population; and to investigate the impact of selected model assumptions –— such as migration or prevalence data from the early years of the epidemic –— on observed discrepancies.

## Methods

### Spectrum file setup

Throughout the article, we will refer to ‘model estimates’ as the outcomes provided by the Spectrum/Estimation and Projection Package (EPP) model. The model version that was used for this study (v5.441) was released in June 2016 [17].

The first step of the analysis was to setup a subnational Spectrum/EPP model based on local demographic, epidemic, and programme data. The EPP component of Spectrum estimates the scale and structure of the HIV epidemic by fitting a model to prevalence data over time provided by the user. A purpose-built Spectrum file was used for the study, based on prospective general population census and cohort data collected in Manicaland province, east Zimbabwe. These data have been described extensively over the past 15 years [18–20]. Briefly, they comprise successive household censuses/surveys and surveys of the main ANCs in 12 sites in three districts of the province. Selection of the sites aimed at capturing a high level of population heterogeneity within the area, as it includes small villages, tea, coffee and forestry estates, and towns. Interviews were followed by HIV tests. Five rounds of data collection were completed between 1998 and 2011. The Spectrum simulation aims to represent the open cohort of household survey participants that have been enumerated in these survey rounds. This primary source of data was used whenever possible; otherwise, the national Zimbabwean 2015 Spectrum file was used. The household cohort data was also combined with the national estimates to calculate women’s fertility rates over time. In a similar fashion, the mortality estimates based on the cohort data were combined with the Coale-Demeny North mortality table to provide life expectancy values over time [21], and the same table was used as a life table. A table showing the main model parameter choices is provided in the supplementary material, http://links.lww.com/QAD/B47. The Spectrum population size was calibrated based on the 2010 household survey population.

The influence of internal migration on the population at subnational levels is critical for the development of robust subnational estimates. As a consequence, it is believed that migration can play a central role in local epidemics, but reliable data may be harder to obtain. This feature can be especially important for urban and rural areas, as the demographic and epidemiological characteristics of outmigrants, inmigrants, and long-term residents may differ (i.e. particularly as HIV prevalence is typically higher in urban areas than in rural areas). Numbers of net migrants and their age structure were calculated using the household census questionnaires, and net migration was assumed to be zero prior to 1998 (the first year of data collection). Spectrum, currently, does not allow for differences in HIV prevalence between migrants and residents.

Estimates for HIV programme statistics (number receiving ART and PMTCT services) for the study sites were based on data from the individual cohort questionnaires (actual PMTCT coverage percentages from rounds 3 to 5, and ART for rounds 4 to 5 were input into the model), except for the types of PMTCT treatment regimens, which were assumed to be the same as those observed at national level.

The default parameters for east Africa were used for the natural history of HIV infection (e.g. average duration of CD4^+^ cell count category, AIDS mortality). These are reviewed and updated by the UNAIDS reference group on estimates, models, and projections [22]. The ratio of fertility of HIV-positive women to fertility of HIV-negative women was derived from the individual Manicaland cohort survey [23,24] and was higher than the national values.

### EPP population and HIV data

The models assumed that there were neither subepidemics nor subpopulations for which stratified data were available. The surveillance data were obtained from the main ANCs (*n* = 76) in the study sites and were grouped by year and location. General population data were taken from the individual Manicaland cohort. Models were fitted using the R-spline variant of the EPP model [25].

### Outcomes of interest

The comparisons between model estimates and data were performed until 2010, the last date for which Manicaland data for the 12 study sites were available.

1. Adult (15– 49 years) HIV prevalence and incidence by sex over time were compared with estimates from the general population cohort.
2. Child (0–14 years) HIV prevalence by single year of age was compared with estimates from a child survey conducted during the most recent round of the Manicaland study.
3. The modelled age structure of the population in 2010 was compared to the age structure of the population measured in the household census. The latter was corrected to take into account under-reporting of children aged less than three within the households by doubling their number, based on the 2010–2011 Demographic and Health Surveys (DHS) estimates [26].
4. Overall mortality and mortality among HIV^+^ individuals were compared with estimates from the general population cohort.
5. Prevalence (among children aged under 18) of double and single (paternal and maternal separately) orphans over time was compared with estimates from the household censuses and to estimates for Manicaland province from the Zimbabwe Demographic and Health Survey [27].

Different scenarios were investigated in the study that aimed to represent different situations of data availability. The main scenario reflects what was done by the national estimation team in Zimbabwe in setting up the 2015 national Spectrum file: surveillance data from the local ANCs were complemented by two estimates of HIV prevalence from general population surveys. In the current application, the 2002 and 2007 rounds of the general population survey were used since 5 years is usually the period between two DHS rounds in Zimbabwe. The sex ratio of HIV prevalence from the 2002 survey was specified in the model postcalibration sex-pattern component.

A second scenario reflected a situation where no general population survey data were available for the area (i.e. only ANC surveillance data was fitted by the model). As HIV prevalence among women attending local ANCs in Manicaland is lower than that amongst pregnant women resident in the same areas [23], this scenario did not include the 20% downward adjustment to ANC HIV prevalence estimates recommended by UNAIDS [28]. A third scenario reflected the case where more general population data are available (observations used from all five surveys spanning 1998 to 2011).

It is thought that the HIV epidemic model estimates are improved when data from early in the epidemic are included. However, these data generally are sparse and often collected from different antenatal clinics or households. The improvement from including this limited additional information was evaluated by inputting data from a small survey conducted in 1994 in ANCs in a relatively urban site close to the main study sites in Manicaland. Different ways of inputting this extra information into the model were explored by varying the assumption about the location of this clinic, providing three alternative scenarios:

A: The ANC is located in a site that has not subsequently provided data (the reality).

B and C: The ANC is located in an area that has provided further surveillance data – the spatially closest study site (a rural area) (B), or the ANC site with the most similar population characteristics (a small town) (C).

In a final scenario, we assumed that there was no migration within the population, and evaluated the impact of this assumption on the model demography and HIV incidence estimates.

In addition to these three scenarios, we conducted sensitivity analyses to investigate the influence of three uncertain parameters: the decrease in HIV transmissibility among individuals on ART, the risk ratio of fertility in HIV-positive women compared with HIV-negative women, and the proportion of HIV-positive individuals in the cohort who were eligible for treatment (according to national guidelines). The influence of the EPP fitting method (three different methods are available [29]) was also tested. The impact of recent changes to mother-to-child HIV transmission probabilities and disease progression among children on estimates of HIV prevalence in children were evaluated by comparing estimates for 2010 produced by the latest version of Spectrum and one released in August 2014. Finally, the specific influence of PMTCT on the child HIV prevalence was evaluated by comparing our main scenario with a specific scenario assuming no PMTCT uptake. For the comparisons between models and data, the Spectrum estimates within the data region of confidence were considered to agree with empirical estimates. Whether a model agreed more or less with data than another model (or better ‘fitted’ data) was measured by calculating the Binomial and Poisson likelihoods of the HIV prevalence and incidence estimates.

## Results

**Fig. 1.**
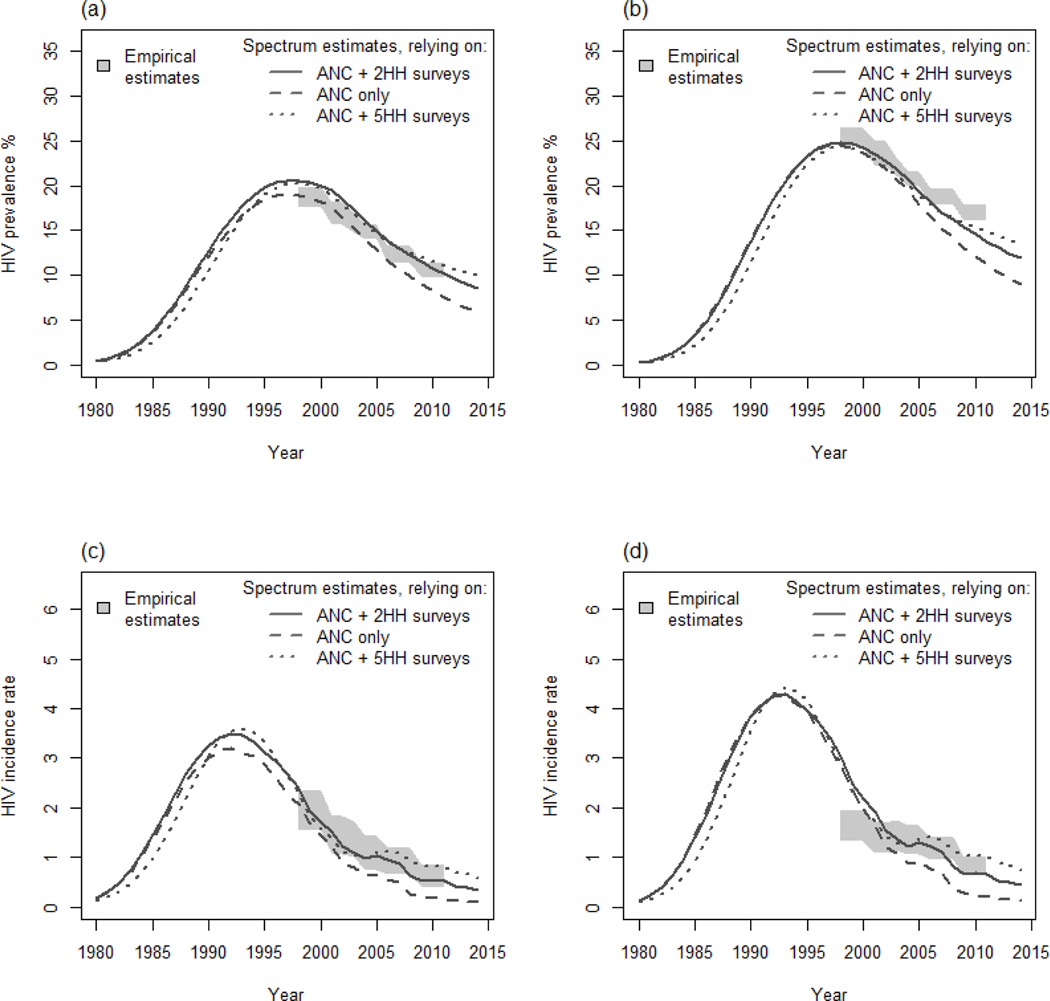
Fitting the Spectrum model to data on the local HIV epidemic in Manicaland, east Zimbabwe: The effects of including data from general population surveys. ANC, ante-natal clinics. HIV prevalence among male (a) and female (b) adults. HIV incidence among male (c) and female (d) adults.

**Fig. 2.**
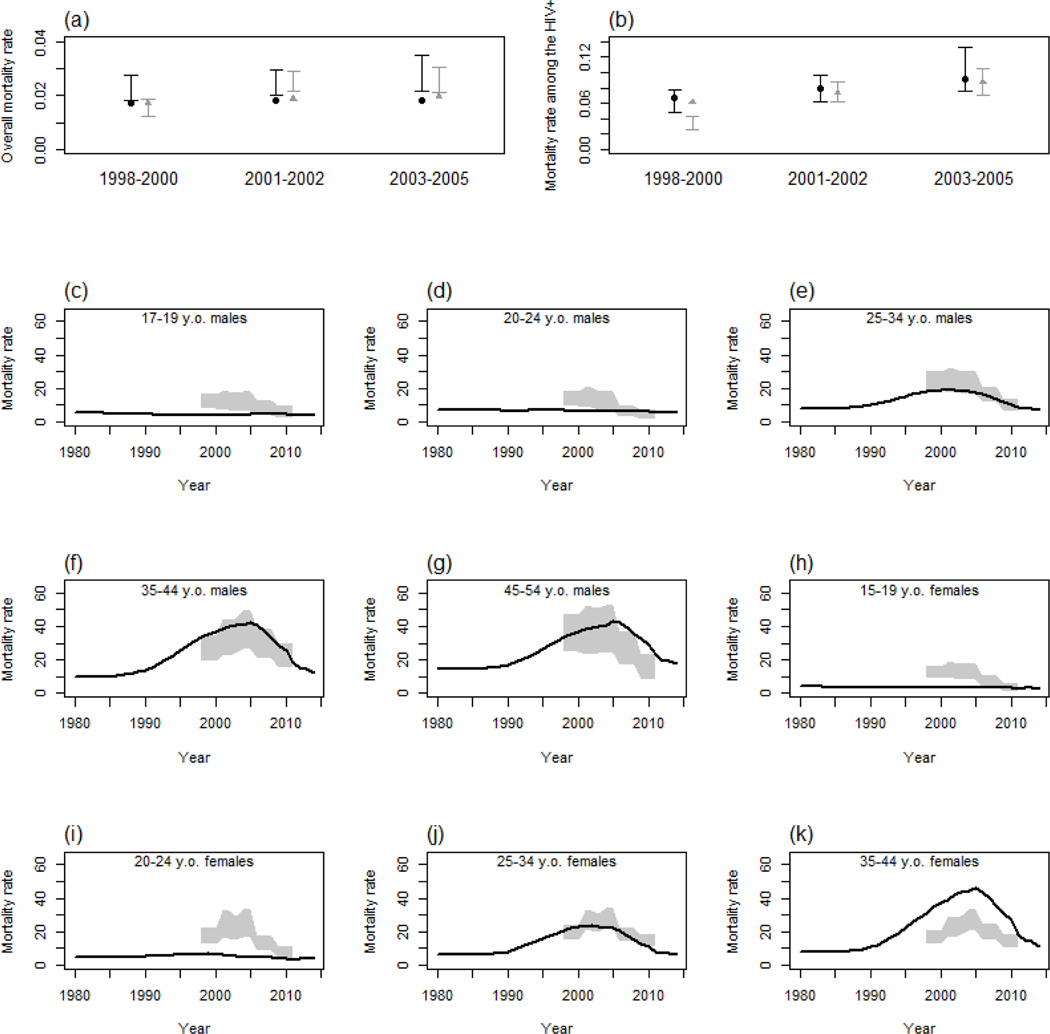
Comparison of model (main scenario) and empirical estimates for all-cause mortality in all adults and HIV^+^ adults. Death rate among all 15–49 males (dark dots), and females (grey triangles) as predicted by Spectrum (a). Death rate among 15+ HIV^+^ males (dark dots), and females (grey triangles) as predicted by Spectrum (b). Overall mortality rates by age among males (c-g), and females (h-k). Error bars and shaded areas represent 95%CI of data.

**Fig. 3.**
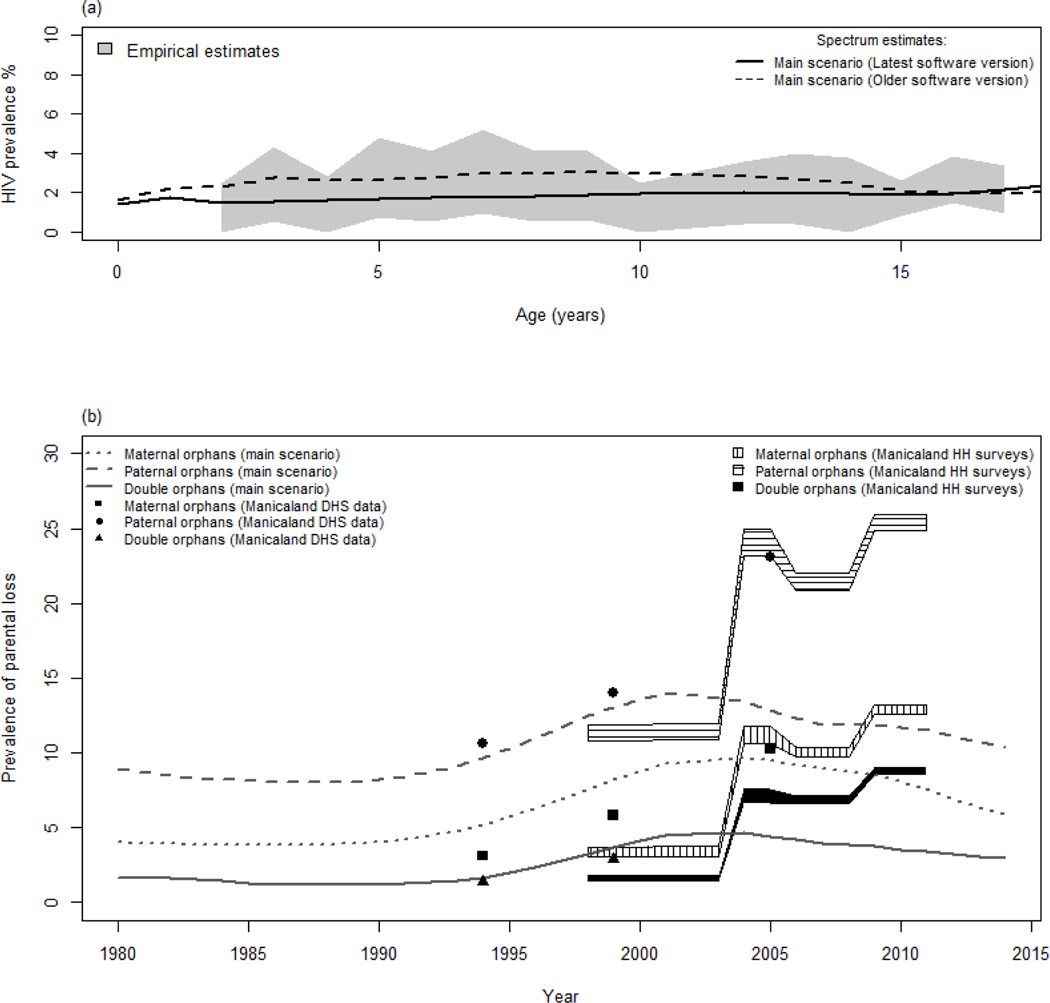
Comparison of model (main scenario) and empirical estimates for the child outcomes. (a) HIV age-prevalence among children in 2010 by model version (current and older). Shaded area shows the 95%CI range for the empirical estimates. (b) Orphan by form of orphanhood over time. Spectrum estimates (lines) compared with the 95% confidence intervals of household survey data estimates (polygons) and Demographic and Health Survey data for Manicaland province, Zimbabwe (squares, dots and triangles).

**Fig. 4.**
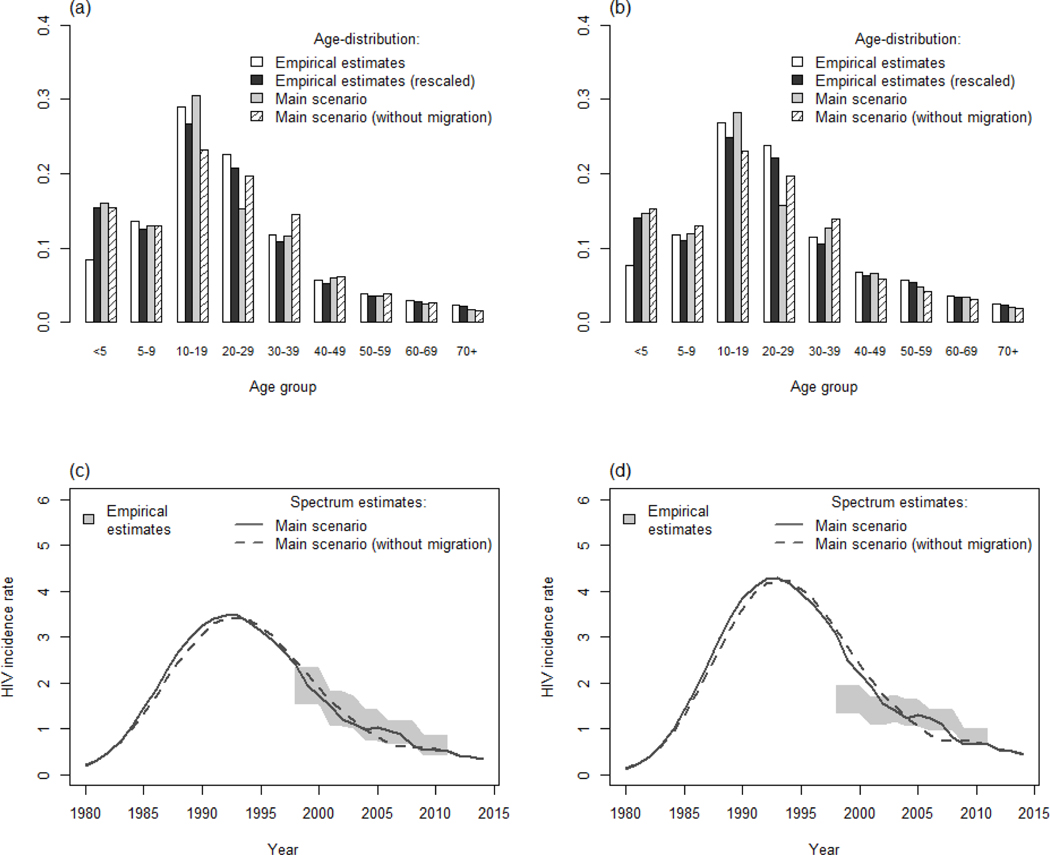
Comparison of model (with and without migration) and empirical estimates for population age structure in 2010. Male (a) and female (b) population age structure in 2010. HIV incidence among male (c) and female (d) adults when migration is included (solid lines) or not (dashed lines).

The main scenario using data from ANC surveillance and two rounds of the general population survey provided accurate estimates of HIV prevalence in adults (Fig. 1a and b). However, the Spectrum-estimated decrease in HIV incidence among women was faster than the empirical estimates (Fig. 1c and d). Slightly lower overall mortality rates were estimated (Fig. 2a), with much lower rates in young adults partially offset by higher rates in older individuals amongst women (Fig. 2) [30]. However, empirical and modelled mortality of HIV-infected individuals were very similar (Fig. 2b). Interestingly, the first three scenarios provided very similar estimates of HIV prevalence among children (not shown), but were ~ 40% relatively lower than estimates provided by a Spectrum model that was released 2 years ago (Fig. 3a) as a result of revisions/updates recently made to the child model in AIDS Impact Model/Spectrum described in elsewhere in this Supplement [11]. The model underestimated the difference in prevalence between maternal and paternal orphanhood in recent years (Fig. 3b). The modelled and empirical population age structures were very similar, except at young adult ages (Fig. 4a and b). Assuming no migration in the model did not increase the discrepancies in population age structure and had only a modest effect on predicted HIV incidence — the predicted decrease in incidence was slightly faster when no migration was assumed (Fig. 4c and d).

The model scenario relying only on ANC surveillance data predicted lower HIV incidence and prevalence rates than the main scenario (Fig. 1), resulting in a poorer likelihood. This was suggested in another analysis of data on pregnant women in the Manicaland study sites [23]. The scenario including five rounds from the general population survey had a similar likelihood than the main scenario and an HIV incidence peak that was later than previously estimated in rural Zimbabwe (~1990) [31] (Fig. 1c and d).

**Fig. 5.**
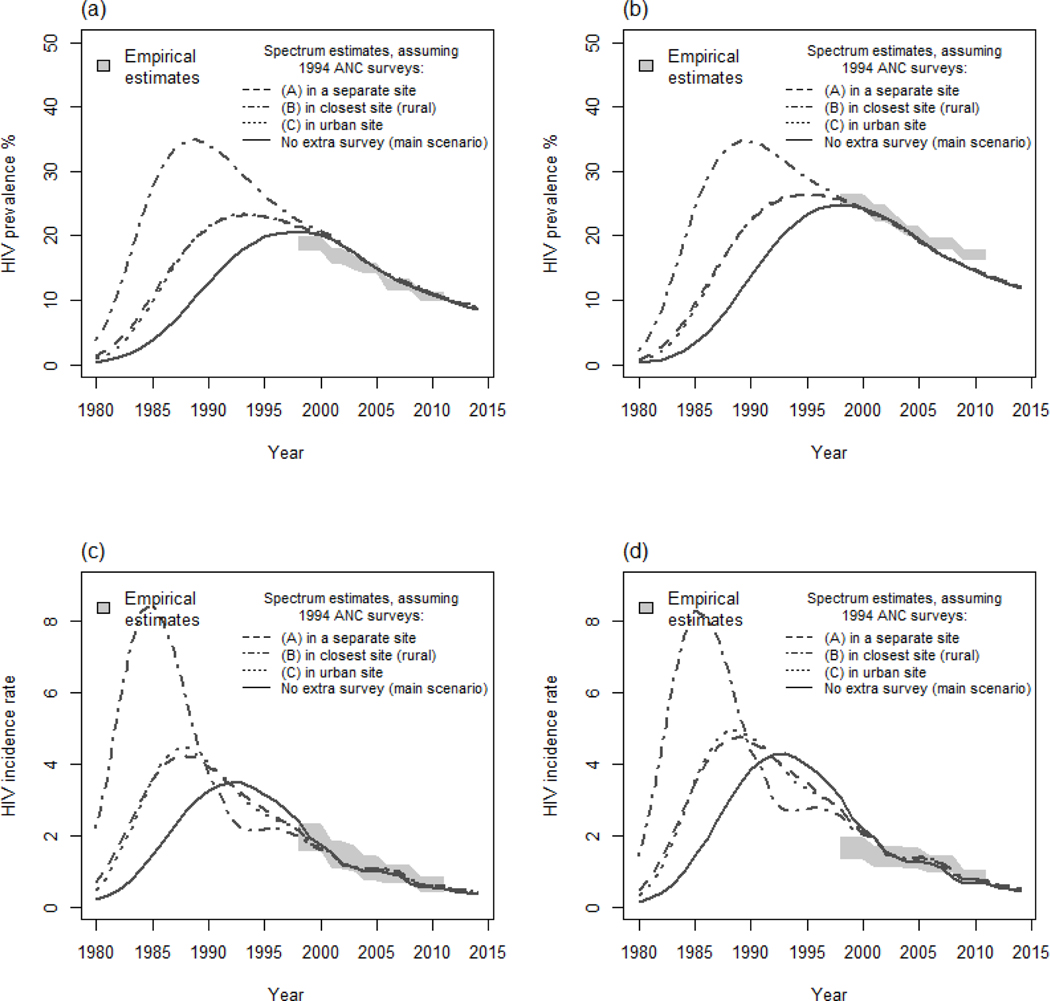
The effect on HIV prevalence and incidence estimates of including data from earlier in the epidemic in Spectrum model fits. ANC, ante-natal clinics. HIV prevalence among male (a) and female (b) adults. HIV incidence among male (c) and female (d) adults.

Adding a single ANC data point at an early stage in the epidemic had a substantial impact on the Spectrum HIV prevalence and incidence estimates before 1998 (Fig. 5). Much higher HIV incidence estimates were obtained when we assumed that the clinic was located in the nearest site that provided data afterwards (B), as this nearest site was rural which suggested that HIV prevalence was very high in a rural area (dash-dotted line). If the HIV prevalence data were assumed to be taken from an additional site (and that this site never reported surveillance data after 1994) (A), the estimated HIV incidence was similar to the case where it was assumed to be located in a small town (C). Overall, inclusion of the extra data point shifted the peak of the epidemic earlier than otherwise was estimated, but the resulting likelihoods were unchanged.

The method chosen to fit HIV prevalence in EPP had an important impact on the estimates (see Supplementary Material, http://links.lww.com/QAD/B47). The ‘EPP classic’ method, not recommended for our situation as we relied on many data points, provided incidence estimates that were slightly closer to the empirical ones, compared with R-spline, but yielded higher HIV prevalence (Fig. S2, http://links.lww.com/QAD/B47). Changing the parameters for which the sensitivity analyses were performed did not have a large impact on the model outcomes, except for modifying the ratio of fertility of HIV-positive women over those of HIV-negative women, which was quite high in the Manicaland data estimates [24]; reducing this ratio to the default values resulted in a drop in the model HIV child prevalence estimates (Fig. S4c, http://links.lww.com/QAD/B47).

The overall impact of PMTCT on the model outcomes was modest, but a significant impact was observed for recent years when HIV prevalence in children was compared with the counterfactual scenario assuming no PMTCT (up to 33% relative reduction for newborns, reflecting recent effects of more effective PMTCT; Fig. S6, http://links.lww.com/QAD/B47). Decreasing ART efficacy in reducing HIV transmission resulted in an earlier peak in HIV incidence, even before its rollout, which is a surprising result (Fig. S3, http://links.lww.com/QAD/B47).

## Discussion

The study reports on an exercise to test the validity of subnational HIV estimates produced by the Spectrum model widely used by countries to generate national estimates. The main scenario reflected the approach followed at national level in Zimbabwe in 2015 where the model was fitted to ANC surveillance data and two rounds of general population survey data on HIV prevalence. Overall, we found reasonable agreement between the Spectrum estimates and empirical data for key epidemiological and demographic indicators but there were several important discrepancies.

The model estimates of HIV prevalence and incidence for men agreed quite closely with the data. However, the model estimates of HIV incidence for women in the early comparison period (1998–2003) were higher and decreased faster than the empirical estimates. This was not expected as mortality in people living with HIV was similar in the model and the data (a rapid decrease in HIV incidence could have resulted from a too large decrease in HIV mortality). Also, all-cause mortality in young adults appeared to be underestimated in the model. This couldhave been corrected by assuming higher values of incidence rate ratios among the adolescents and young adults. However, the Spectrum mortality rates among young adults were only slightly higher than the national estimates obtained in the demographic and health surveys [26,32–34]. Improved methods for estimating orphan levels have been included in Spectrum recently [35]. Here, the model estimates of paternal and maternal orphan prevalence initially matched and were higher than the empirical data. However, from the mid-2000s, the model estimates underestimated all forms of orphanhood [27], partly because of the underestimation of the mortality described earlier, but also because the increase that was observed in both cohort and DHS data between 1999 and 2005 was remarkable (Fig. 3).

The estimates of age-specific HIV prevalence among children decreased in the most recent Spectrum versions, reflecting the changes in the paediatric model [11], and were even more in line with the data. The current Spectrum software appear robust as there was little change in the estimates when additional data were included in the model. Interestingly, a similar model based on national data (and an earlier version of the Spectrum software [17]) estimated significantly higher HIV prevalence rates for these age groups [36].

A small number of generalized epidemic countries (two in 2015, all located in sub-Saharan Africa) still rely on ANC data only for their national estimates. We found poorer agreement when the survey data were omitted for Manicaland, although this may have been affected by local patterns of service use which resulted in lower HIV prevalence in women attending local ANCs than in pregnant women actually resident in the clinic catchment areas [23]. Adding HIV prevalence data from general population surveys modestly improved the model likelihood.

Inconsistencies observed between the model and empirical estimates of epidemiological and demographic outcomes reflect shortcomings in the data as well as possible limitations in the Spectrum model. For example, under or overascertainment of deaths in the Manicaland cohort could have contributed to discrepancies in the mortality estimates. Data were not collected on the PMTCT treatment regimens used by individual women in the study population, so we assumed that these were in line with national guidelines. Therefore, differences in local implementation could have contributed to discrepancies in HIV prevalence in children and in orphan prevalence. Inclusion of data from 1994 for a site close to the study areas in Manicaland showed that Spectrum estimates of HIV trends can be sensitive to inclusion of data from early in an epidemic and yielded an earlier peak in HIV incidence. No epidemic data were available for the Manicaland study sites themselves before 1998, which may have resulted in a delayed estimate of the timing of peak in HIV incidence.

There is growing interest in using the Spectrum model to produce subnational estimates [4,5]. Importantly, the model does not provide uncertainty ranges of the outcomes that were put forward in our analysis, which could be important when applied to subnational epidemics. The results of this validation exercise suggest that Spectrum can provide reasonable local estimates where detailed local-specific data on demographic, epidemiological, and programmatic model inputs are available. However, the unavailability of subnational information, especially epidemiological data, may force modellers to use national data which could produce biased estimates. However, our sensitivity analysis did not identify any specific demographic variables that had dramatic effects on model outcomes. Moreover, the most recent empirical local-level data that were used in the analysis were collected in 2010, and we were not able to study the stability of the discrepancies over more recent years. Another round of data collection in Manicaland was performed after this period, but only within a subsample of the original study sites and including it was considered likely to lead to inconsistent model inputs.

The current Spectrum model enables the user to input levels of net migration by 5-year age group. For national estimates of generalized epidemics, distinguishing HIV prevalence levels between inmigrants, outmigrants, and residents has not been considered necessary because, in most instances, numbers of international migrants are small. However, for subnational estimates, population turnover because of migration may be much greater. Furthermore, outmigrants and, especially, inmigrants can have very different levels of HIV prevalence compared to long-term residents [37,38]. In rural areas, many inmigrants will come from towns and other centres of labour migration where rates of HIV infection, typically, are much higher. The effects of this may be offset if outmigrants also have higher HIV prevalence than nonmigrants, which has been observed in some African countries [39]. However, where recent inmigrants represent a significant fraction of the population at a given time, failure to account for differences in HIV prevalence within age groups could cause overestimation of incidence of new infections in rural areas. Similarly, replacement of outmigrants with high HIV prevalence with inmigrants with lower HIV prevalence could result in underestimates of HIV incidence in towns, mines, and commercial estates. Differences in temporal trends in HIV infection between migrants and resident populations could distort estimates of subnational trends. In the current study, these effects may have been ameliorated by the mixture of small towns, estates, and rural villages within the study sites in Manicaland, Zimbabwe. Also, the HIV epidemic in Zimbabwe is often considered to be geographically more homogenous than in surrounding countries [12]. The HIV prevalence of inmigrants within rural survey sites in Manicaland is shown in the Supplementary Material (Figure S7, http://links.lww.com/QAD/B47).

Previous Spectrum/empirical data comparisons have been conducted [4–7]. However, in most cases, these have been piecemeal assessments with only a small number of selected outcomes being examined (e.g. mortality). For example, in a study in Northwestern Tanzania, Spectrum estimates for mortality trends were similar to empirical estimates [4] but no other model outcomes were evaluated; importantly, national-level demographic and epidemic data were used in the model. Strengths of our analysis are its comprehensive and holistic approach and use of subnational data for key model inputs.

HIV infection rates and outcomes vary significantly between places so it is essential that subnational epidemic estimates are produced. However, validation of subnational Spectrum estimates with locally specific empirical data is an important step before these estimates can be relied upon by decision-makers in formulating policy and allocating resources.

## Supplementary Material

UNAIDS supplemental material
