# Supplementary material for: Empirical validation of the UNAIDS Spectrum model for subnational HIV estimates: case-study of children and adults in Manicaland, Zimbabwe
Source: AIDS. Author manuscript; Available in PMC 2023 Nov 21. (PMC10660499; doi:10.1097/QAD.0000000000001418)
Supplement: UNAIDS supplemental material [file NIHMS1931610-supplement-UNAIDS_supplemental_material.pdf]

Empirical Validation of the UNAIDS Spectrum Model for Subnational HIV Estimates: Case-Study of Children and Adults in Manicaland, Zimbabwe

Romain SILHOL[1], Simon GREGSON[1,2], Constance NYAMUKAPA[1,2], Mutsa MHANGARA[3], Janet DZANGARE[3], Elizabeth GONESE[4], Jeffrey W EATON[1], Kelsey K CASE[1], Mary MAHY[5], John STOVER[6], Owen MUGURUNGI[3]

[1] Imperial College London, Infectious Disease Epidemiology, London, United Kingdom

[2] Biomedical Research and Training Institute, Avondale, Harare, Zimbabwe

[3] AIDS and TB Unit, Zimbabwe Ministry of Health and Child Welfare, Harare, Zimbabwe

[4] Division of Global HIV and TB, U.S. Centers for Disease Control and Prevention, Harare, Zimbabwe

[5] Programme Branch, UNAIDS, Geneva, Switzerland

[6]Avenir Health, Glastonbury, Connecticut, United States

Supplementary material

Supplementary Table 1: Spectrum model parameters

Parameters	Value/Comments
**Demography**	
First-year population (1970)	1987 Zimbabwe Inter-Censal Demographic Survey
Total fertility rate	Manicaland round 1 to 5 household surveys estimated TFR were combined with UN Pop national trend.<br>1970: 6.7. 1980: 6.3. 1990: 4.67. 2000: 3.6. 2010: 3.6
Age-distribution of fertility (%)	Manicaland ANC surveys were used<br>15-19: 22.5%. 20-24: 33.5%. 25-29: 22.5%. 30-34: 12.8%. 35-39: 6.5%. 40+: 2.2%.
Life-expectancy (without HIV)	Calculated by triangulating the Manicaland estimates of 45q15 throughout the study period and the Coale Demeny North mortality table.<br>1970: 47.5(F), 42(M). 1980: 52(F), 46(M). 1990: 59(F), 53(M). 2000: 59(F), 57(M). 2010: 62(F), 53(M)
Migration	The number and age of in/out migrants between rounds was extracted from the data, and net-quantities were provided to Spectrum*
**Programme statistics**	
PMTCT	Overall coverage % was input into Spectrum, using the round 3 to 5 individual data, and the type of treatment regimen was determined using the national estimates*.
ART	The proportions of HIV+ individuals on ART were based on self-reports in the Manicaland general population surveys, then it was assumed that 50%* of HIV+ individuals were eligible. Quantities were interpolated to provide estimates for each year, from 10% in 2006 to 80% in 2015.*
Child treatment	Round 5 children questionnaire was used to determine the ART coverage (23%)
**Transition parameters**	
Years in CD4 category, HIV mortality with/without ART, treatment effects…	UNAIDS default parameter settings
Reduction in HIV infectivity when on ART	70% *
Ratio of fertility of HIV+ women to fertility of uninfected women	Based on Terceira et al. 2003.*<br>15-19: 3.4. 20-24: 0.9. 25-29: 0.7. 30-34: 35+: 0.6
**HIV data**	
Gender HIV prevalence ratio	In 1999 : 1.36 (25.5/18.5)<br>In 2002 : 1.39 (23.5/16.9)<br>The empirical 1999 gender HIV prevalence ratio was only used when 5 rounds of general population HIV prevalence were inputted to the model. The value for 2002 was used otherwise

*a sensitivity analysis is provided within this supplementary document

Supplementary Figure 1: Spectrum (main scenario) and empirical population age-structure estimates over time.

The discrepancies between model (dark) and rescaled data (grey) over time are similar to those observed in 2010 (see main manuscript). The empirical data was simply rescaled by assuming an original 50% under-reporting of the number of children aged under 4 in the household.

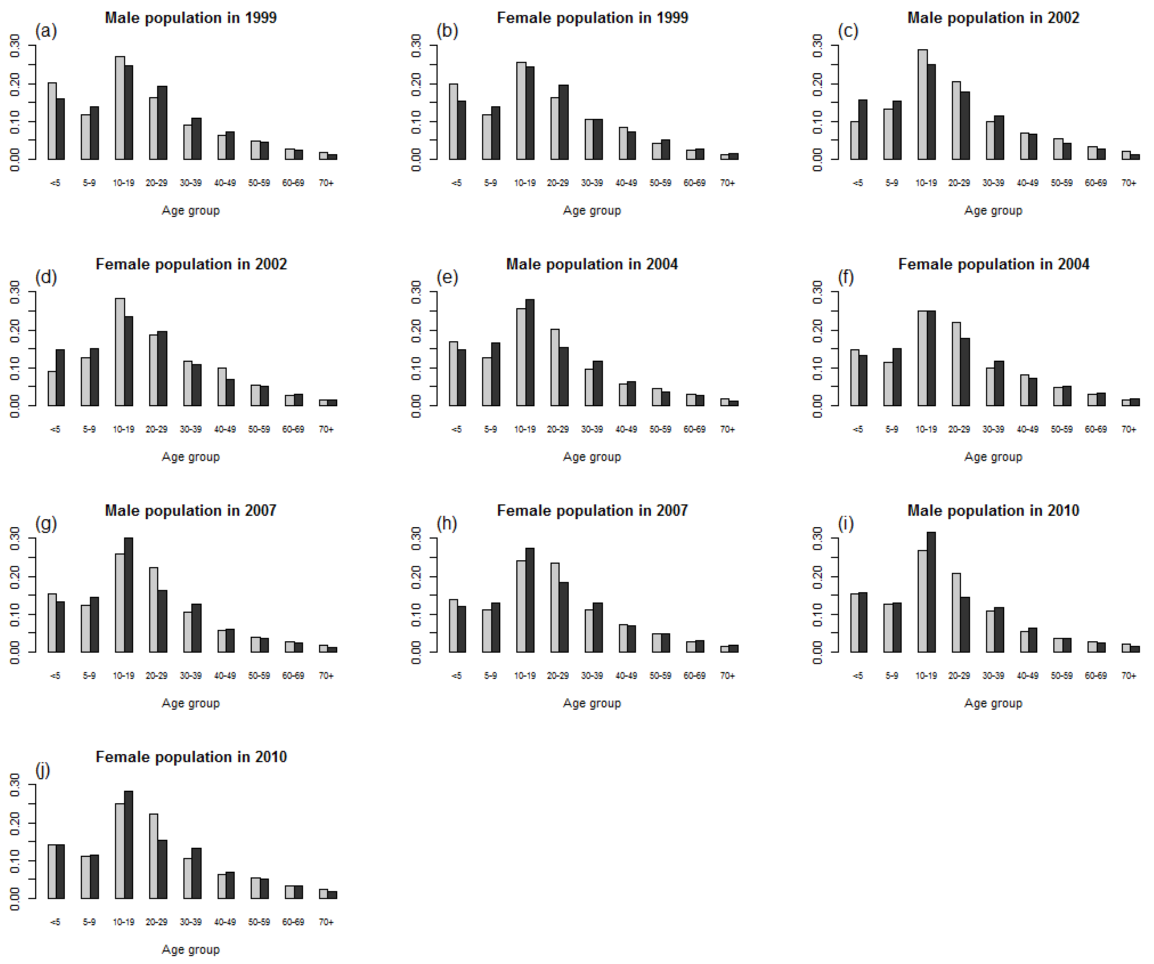

Supplementary Figure 2: Alternative methods for fitting EPP to HIV prevalence data. HIV prevalence among male (a) and female (b) adults. HIV incidence among male (c) and female (d) adults.

The R-spline method for fitting EPP is recommended by UNAIDS for our situation since we inputted a lot of data points. However, the outputs obtained from the preferred alternative methods (R-trend) appeared to provide similar matches to the data, whereas HIV incidence estimates provided by the EPP classic method were significantly higher.

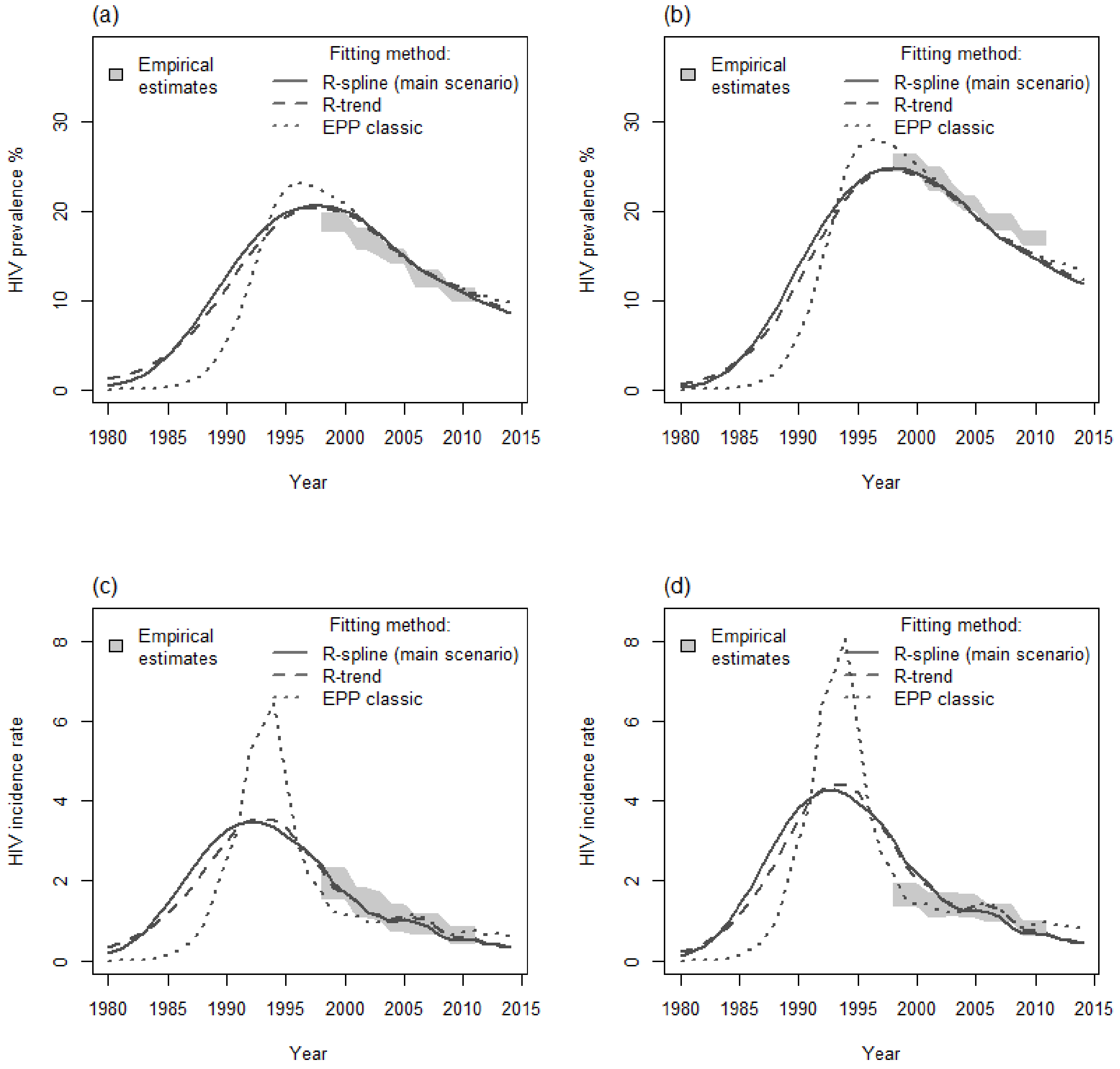

Supplementary Figure 3: The effect of varying the extent to which ART reduces HIV transmissibility. HIV prevalence among male (a) and female (b) adults. HIV incidence among male (c) and female (d) adults. ART efficacy was varied from 50% to 95% but had no influence on the predicted HIV incidence after 2000, and a significant one before that, while the reverse was expected.

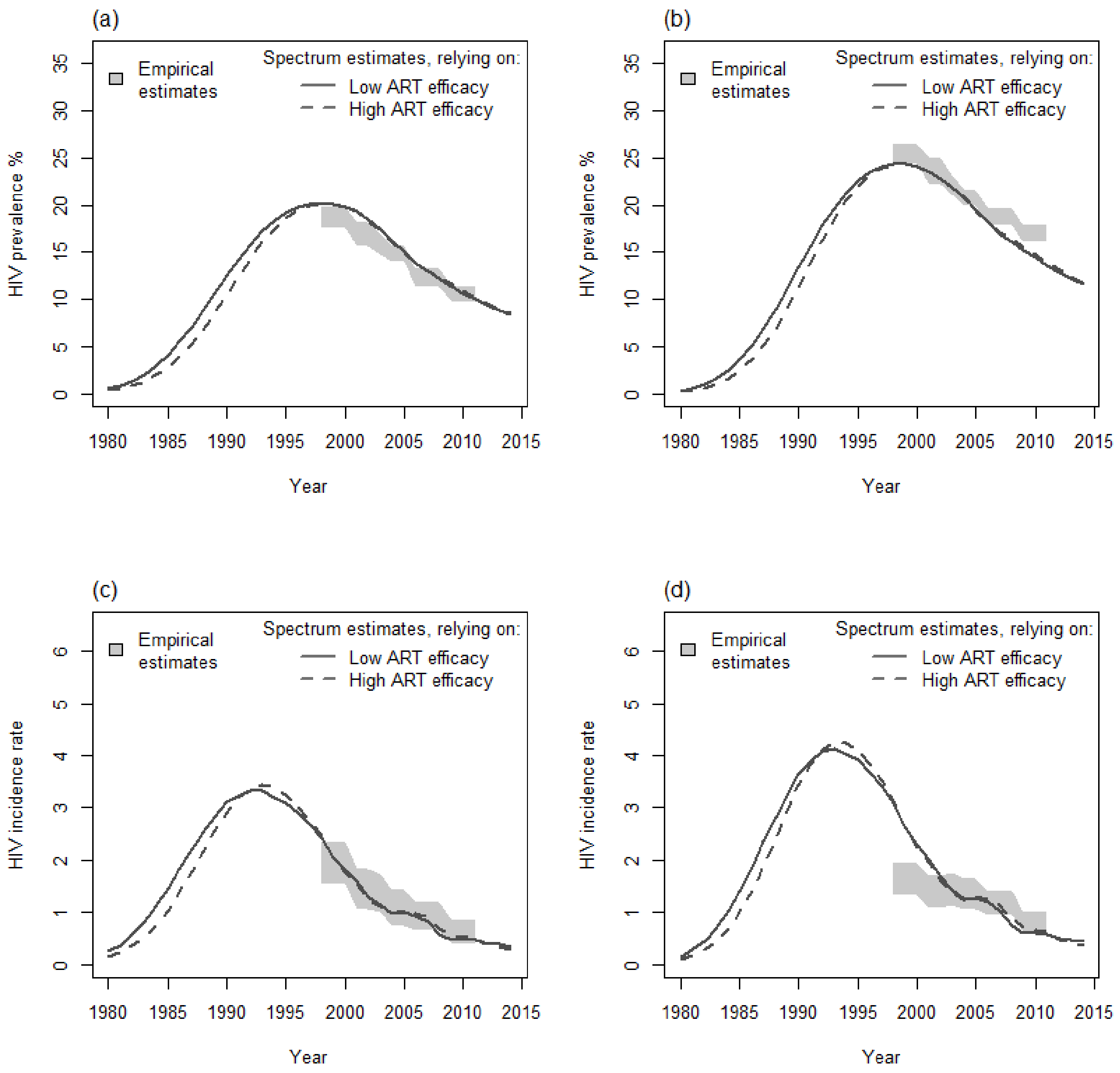

Supplementary Figure 4: Varying the risk-ratio of fertility in HIV-positive women versus uninfected women at all ages. HIV incidence among male (a) and female (b) adults. HIV prevalence among children in 2010 (c). Dashed lines are the model default values: RR = 1.20 for 15-19 y.o., decreased to 0.47 for the 45-49 y.o. Plain lines are the Manicaland estimates: RR = 3.5 for 15-19 y.o, decreased to 0.95 for 20-24 y.o. and then to 0.64 (see table 1).
Assuming higher fertility of HIV+ women (as was done in the scenarios in the main paper) significantly increases HIV prevalence in children (c), but does not have an impact on the HIV incidence estimates (a-b).

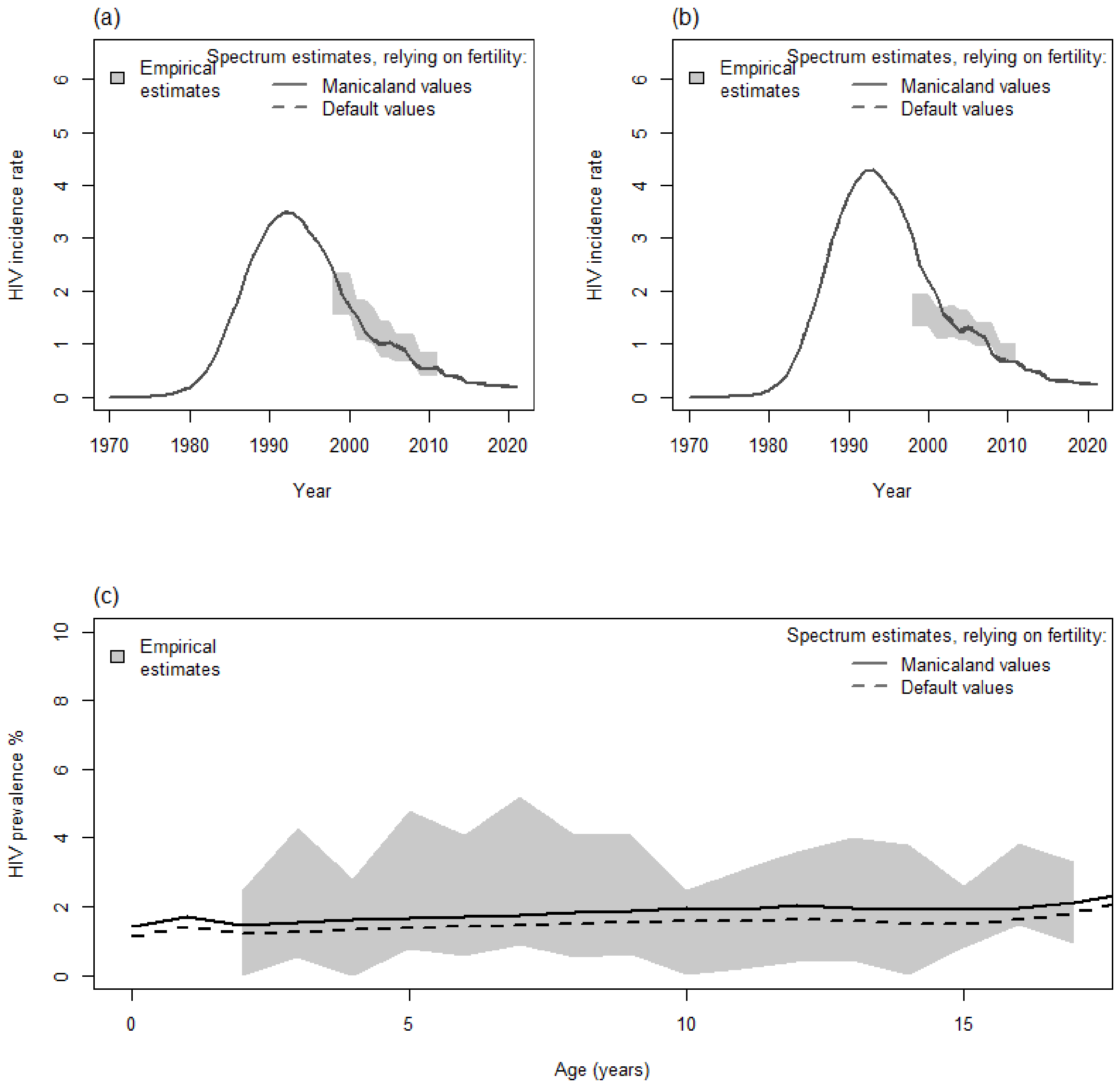

Supplementary Figure 5: Proportion eligible for ART (CD4 count < 200) among HIV-positive adults in the surveys.

HIV prevalence among male (a) and female (b) adults. HIV incidence among male (c) and female (d) adults. We assumed that the proportion of infected individuals who were eligible for ART remained constant over time, except for 2010 when the threshold for eligibility was increased to CD4=350. Lower bound: 35% of infected individuals eligible. Upper bound: 65% eligible. Assuming an actual low coverage of ART in the population slightly increased the estimates of HIV prevalence and incidence before the peak of the epidemic, which was surprising.

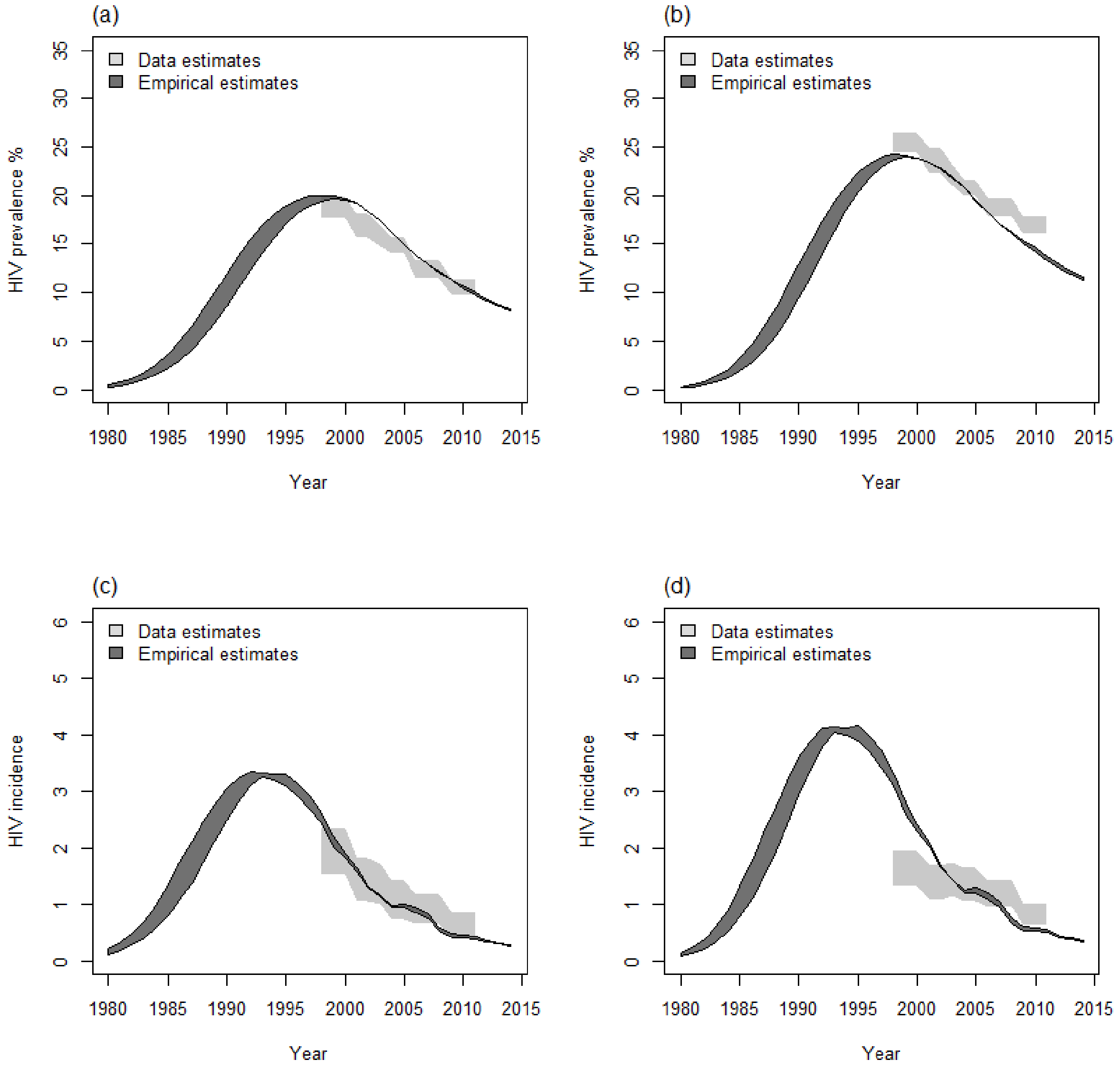

Supplementary Figure 6: Impact of the scale-up of PMTCT services on age-specific HIV prevalence in children as at 2010. HIV prevalence among children in 2010. The impact of PMTCT was tested assuming no uptake throughout a specific scenario (dashed line). The overall impact of PMTCT was modest but a significant impact was observed at younger ages reflecting increased coverage of more efficacious regimens in the recent years. Removing the effects of PMTCT services did not alter the HIV incidence estimates (not shown).

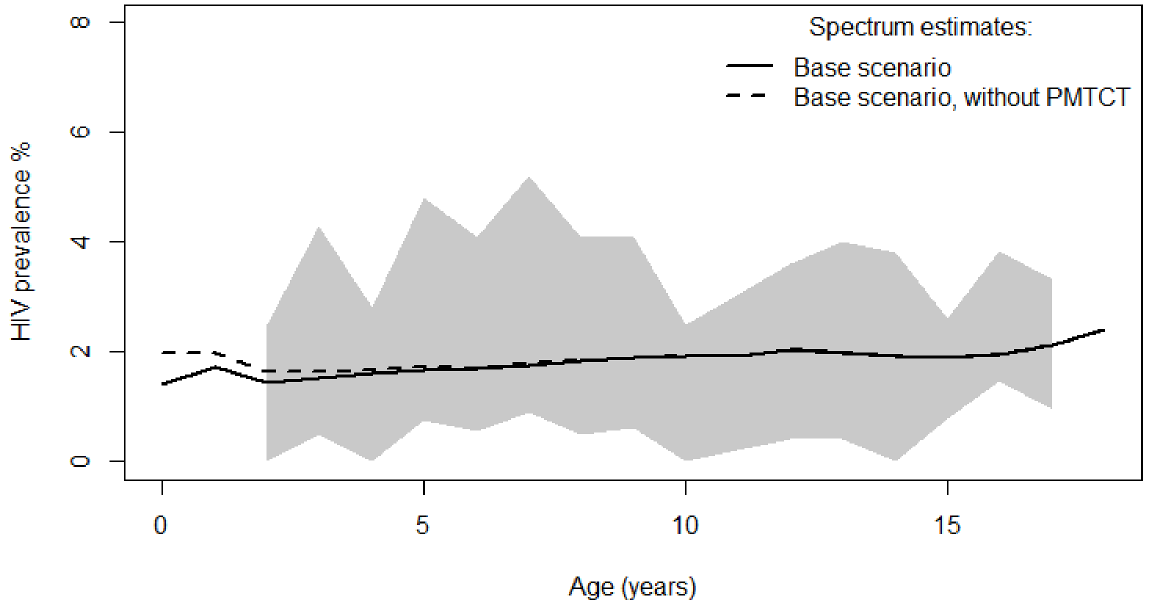

HIV prevalence of in-migrants within Manicaland survey villages

Migration is a phenomenon that influences the dynamics of HIV epidemics but that can be difficult to model. In Manicaland, migration rates fluctuated considerably over time due to socio-economic instability, so it is possible that migration contributed to the differences in model and empirical estimates of trends in HIV incidence found in this study.

Overall, out-migrants had similar HIV prevalence to non-migrants (1). However, in 1998, male in-migrants had similar HIV prevalence to male non-migrants (21%), while female in-migrants at this time had higher prevalence (29% versus 25%). Infection rates in both sexes subsequently fell more rapidly in in-migrants than in non-migrants (2010: males – 8% in in-migrants versus 13% in non-migrants; females – 15% versus 17%) (unpublished data). In other rural settings, the characteristics of out-migrants would matter more if they were a more important component of the demography dynamics.

Figure S7 reports the prevalence of residents and in-migrants in the rural villages of the Manicaland cohort. In-migrant females in these areas generally had higher HIV prevalence but there was no difference for males.

Supplementary Figure 7: HIV prevalence of in-migrants in the villages of the Manicaland cohort. HIV prevalence among residents and in-migrants of villages among males (a) and females (b), standardised for age and location. Error bars represent mean 95% CI. Migration was defined as moving into an area in the 3 years prior to the interview.

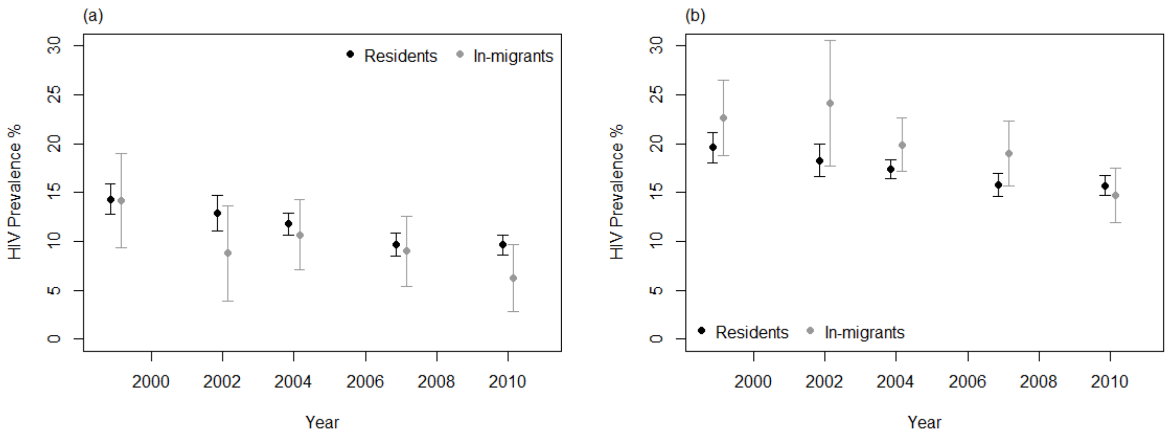

Supplementary Figure 8: ANC and general population HIV prevalence data in EPP. Red dots and intervals: HIV prevalence data from general the 2002 and 2007 general population survey rounds (n=6483, n=10463). Green dots and curves: HIV prevalence data from the 12 main ANCs of the study sites from 1998 to 2011 (n=76 data points)

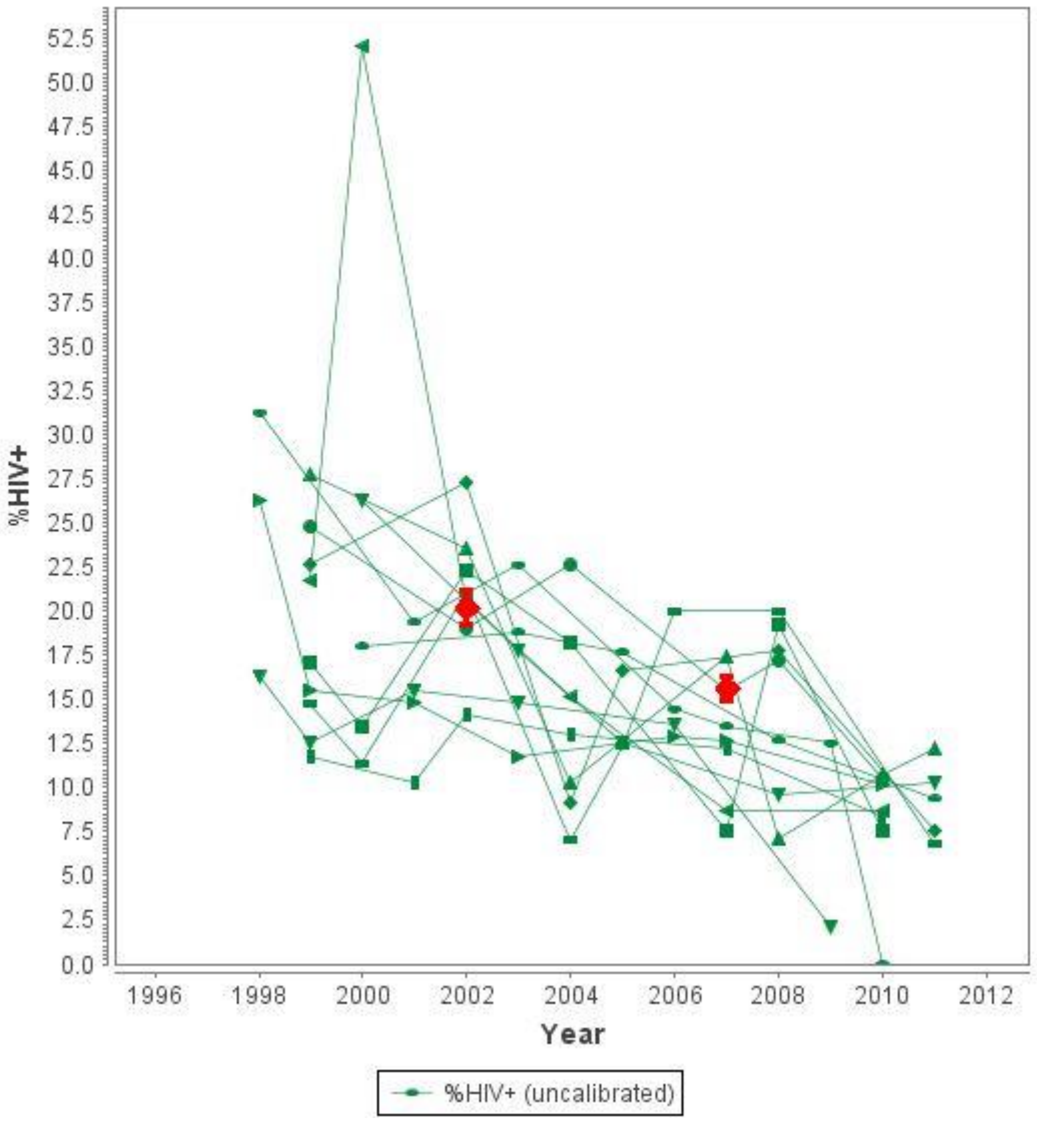

References

1. Mundandi C, Vissers D, Voeten H, Habbema D, Gregson S. No difference in HIV incidence and sexual behaviour between out-migrants and residents in rural Manicaland, Zimbabwe. Trop Med Int Health. 2006;11(5):705-11.
